# The Food Production Environment and the Development of Antimicrobial Resistance in Human Pathogens of Animal Origin

**DOI:** 10.3390/microorganisms5010011

**Published:** 2017-03-14

**Authors:** Manjusha Lekshmi, Parvathi Ammini, Sanath Kumar, Manuel F. Varela

**Affiliations:** 1 QC Laboratory, Harvest and Post Harvest Technology Department, ICAR-Central Institute of Fisheries Education (CIFE), Seven Bungalows, Versova, Andheri (W), Mumbai 400061, India; manjusha@cife.edu.in (M.L.); sanathkumar@cife.edu.in (S.K.); 2 CSIR-National Institute of Oceanography (NIO), Regional Centre, Dr. Salim Ali Road, Kochi 682018, India; parvathi@nio.org; 3 Department of Biology, Eastern New Mexico University, Portales, NM 88130, USA

**Keywords:** antibiotic, growth promoter, pathogen, resistance, zoonosis

## Abstract

Food-borne pathogens are a serious human health concern worldwide, and the emergence of antibiotic-resistant food pathogens has further confounded this problem. Once-highly-efficacious antibiotics are gradually becoming ineffective against many important pathogens, resulting in severe treatment crises. Among several reasons for the development and spread of antimicrobial resistance, their overuse in animal food production systems for purposes other than treatment of infections is prominent. Many pathogens of animals are zoonotic, and therefore any development of resistance in pathogens associated with food animals can spread to humans through the food chain. Human infections by antibiotic-resistant pathogens such as *Campylobacter* spp., *Salmonella* spp., *Escherichia coli* and *Staphylococcus aureus* are increasing. Considering the human health risk due to emerging antibiotic resistance in food animal–associated bacteria, many countries have banned the use of antibiotic growth promoters and the application in animals of antibiotics critically important in human medicine. Concerted global efforts are necessary to minimize the use of antimicrobials in food animals in order to control the development of antibiotic resistance in these systems and their spread to humans via food and water.

## 1. Introduction

Antimicrobial compounds, which include antibiotics and chemicals intended to kill or halt the proliferation of unwanted bacteria, have historically changed the perspective of controlling microorganisms. In the beginning of the 20th century, Paul Ehrlich’s incessant search for antimicrobials resulted in the synthesis of chemical compounds salvarsan and neosalvarsan which were widely used to treat the sexually transmitted disease syphilis caused by *Treponema pallidum* [1]. The first antibiotic penicillin was considered a “magic bullet” against microbial infections in the last century. The discovery of penicillin was soon followed by sulfa drugs, gramicidin, streptomycin and an array of antibiotics belonging to different chemical classes and with different biological activities. Infectious diseases were thought to have been controlled, and the magic of antibiotics continued for a specific period of time during the mid-20th century, called the “golden era of antibiotic chemotherapy” [2].

With advances in microbiological, molecular biological and analytical techniques, numerous antibacterial compounds could be identified, purified or even synthesized for human use. Thus, antimicrobials have been the immediate choice in the event of any microbial infections of humans or animals. Regardless of the need for their application, these powerful compounds were used to rid microorganisms of biotic and abiotic surfaces, cure infections in humans and animals and even to preserve foods [3]. However, bacterial pathogens soon exhibited the signs of surviving the lethal effects of antimicrobials, and the first penicillin-resistant *Staphylococcus* was reported soon after the introduction of penicillin in 1946, although penicillinases were discovered considerably before the discovery of penicillin itself, suggesting the occurrence of natural reservoirs of antimicrobial resistance genes [4,5]. In the clinical settings, inappropriate use accounts for 20%–50% of the antibiotics consumed [6]. These include the use of antimicrobials for viral infections, the prescription of broad-spectrum antibiotics, non-compliance to a treatment regimen by the patient, and incorrect dosage or duration [7,8].

Over the years, antibiotics have also been used in animal husbandry and aquaculture for growth promotion, feed efficiency, prophylaxis, as well as in the treatment of infections. Indiscriminate use of antibiotics for purposes other than treatment of infections has resulted in the emergence of antibiotic-resistant pathogens in food production environments, and such pathogens can cause infections in humans that are difficult to treat or control. Bacteria may develop resistance due to the exposure to sub-lethal levels of antibiotics in their surroundings, or alternatively, bacteria may directly acquire resistance mechanisms from other bacteria via DNA transfer mechanisms. The use of antibiotics in food and agriculture has direct as well as indirect effects on the development of antibiotic resistance by bacteria associated with animals which can enter into the food chain through meat and fish. According to the United States Centers for Disease Control and Prevention (CDC), more than two million people in the United States are infected by antibiotic-resistant bacteria leading to the death of about 23,000 people annually [9]. In Europe, an estimated 25,000 deaths are due to antibiotic-resistant bacteria [10]. In the United States, nearly 80% of the antibiotics produced are used in animal husbandry [11].

The global increase in the population consequently demands that the production of meat-yielding animals and birds is proportionately increased as well. The introduction of the fluoroquinolones in the poultry industry in the United States led to *Campylobacter* quickly gaining resistance to these antimicrobial agents. It is now well established that the use of antibiotics in livestock and aquaculture results in the development of resistance in human pathogens directly or indirectly, as susceptible bacteria die, leaving behind resistant variants which in turn predominate [12]. Human infections by such resistant bacteria become either untreatable or the treatment becomes prolonged, complicated and expensive [13]. The efficacy of many popular and effective antibiotics is in peril, a situation that can potentially lead to severe crisis in the treatment of infectious diseases in the near future. The morbidity and mortalities due to various infectious diseases in densely populated, low-income countries are clearly increasing, and their treatment is critically affected by the development of antibiotic resistance among pathogenic bacteria. The use of antibiotics for short-term gains has inflicted a long-lasting negative impact on human health and in the environment [14].

## 2. Mechanisms of Bacterial Antibiotic Resistance

Over the course of their evolution, bacteria have developed several known key cellular biological mechanisms that confer resistance to antimicrobial agents [15]. These important bacterial resistance mechanisms are briefly considered below.

One of the earliest known resistance mechanisms involved the enzymatic inactivation of antimicrobial agents [16]. These enzymes will hydrolytically metabolize antimicrobial agent substrates into inactive metabolic end-products that in turn do not kill or inhibit the growth of bacteria. The first case of enzymatic inactivation involved the disarming of the penicillins and other β-lactam antimicrobials with penicillinases, known also as β-lactamases [17]. Disturbingly, enzyme variations emerged in which both the catalytic activities and substrate specificities were greatly enhanced, and these enzymes were collectively called extended spectrum β-lactamases (ESBLs) [18]. These ESBLs constitute a significant challenge both in the agricultural as well as in the human and veterinarian clinical settings [19]. Other hydrolytic enzymes include esterases, which inactivate certain macrolide antimicrobials [20,21]. On the other hand, microbial enzymes may biochemically modify antimicrobial agents by attaching adducts in order to render the modified drugs unable to kill or inhibit the growth of bacteria. For example, acetyltransferases attach acyl groups to aminoglycoside antibiotics [22].

Another bacterial resistance mechanism involves the modification of the target by which the antimicrobial agents mediate their inhibitory effects. Such drug resistance mechanisms are referred to as target alteration or modification [23]. Extensively studied bacterial targets include cellular machinery for the synthesis of proteins, such as the ribosome [24], as well as the syntheses of bacterial cell walls, such as transpeptidases [25], or of nucleic acids, such as DNA gyrase [26] and RNA polymerase [27]. A well-known example of this type of resistance mechanism includes those determinants for quinolone and fluoroquinolone resistances in which DNA gyrase, the target for these antimicrobials, is subtly altered, often by point mutation, such that the antimicrobials cannot bind to these target variants [28]. Consequently, the bacterium is able to grow even in the presence of clinical concentrations of the quinolones and fluoroquinolones [29].

A third resistance mechanism involves blocking of the antimicrobial target, i.e., target protection [30,31]. A well-studied bacterial mechanism of this type includes resistance to the tetracyclines, a class of protein synthesis inhibitors that bind to the 30S subunit of the ribosome, in which the tetracycline binding site on the ribosome is protected by small peptides that prevent the antibiotic from binding to its target [30,31]. A newer synthetic derivative of the tetracyclines, a glycylcycline called tigecycline, overcomes the effects of this protection mechanism [32], an effect that is unfortunately reversed by active drug efflux transporters that confer tigecycline resistance [33].

A fourth bacterial resistance mechanism relies on the ability of the bacterium to reduce the entry of the antimicrobial agent into the cytoplasm [34]. This mechanism is referred to as drug permeability reduction and prevents access of antimicrobial agents to their intracellular drug targets, thus conferring antimicrobial resistance [35,36]. A well-known and extensively studied drug permeability reduction mechanism involves porin channels which reside in the outer membrane of Gram-negative bacteria [37]. These porins may be either downregulated or defective such that their permeation activities are severely reduced, in any case greatly curtailing access to intracellular targets [36]. Bacteria harboring these determinants are essentially resistant to clinically important antimicrobials such as the aminoglycosides, the β-lactams, the fluoroquinolones and the chloramphenicols [37]. Alternatively, additional elements in the cell walls may function in synergy with the outer membrane proteins to mediate the active efflux of multiple antimicrobials, a system that is known as a multipartite complex consisting of the outer membrane protein, a periplasmic membrane fusion protein, and an inner membrane transporter [36].

The last bacterial mechanism considered here involves the active efflux of antimicrobial agents from the bacterial cell, where most, if not all, of the drug targets reside [38,39]. The efflux of multiple antimicrobials from the intracellular location of the bacterium effectively reduces the drug concentrations and confers resistance to antimicrobials [40,41]. These resistance mechanisms involve several major and large classes of transporter protein superfamilies [42]. The ATP-binding cassette (ABC) transporters use the biological energy of ATP hydrolysis to mediate the extrusion of antimicrobial agents from the bacterial cell [43]. The remaining superfamilies use either passive or secondary active transport modes to drive drug and multidrug efflux in bacteria. Secondary active transport systems use ion gradients generated by respiration as the driving energy force required for active drug efflux [44,45], and include the resistance-nodulation-cell division (RND) superfamily [46], the small multidrug resistance (SMR) superfamily [47], the multidrug and toxic compound extrusion (MATE) superfamily [48,49], and the major facilitator superfamily (MFS) of solute transporters [50], such as those seen in the *Enterobacteriaceae* bacterial family, the cholera pathogen *Vibrio cholerae* [51] and *Staphylococcus aureus* [52,53], as well as in *Salmonella enterica* [54]. Although the crystal structures have been solved for only a few key multidrug efflux pumps [50], these MFS multidrug efflux pumps make good targets for modulation, such as inhibition [55,56], in order to restore the clinical efficacy of antimicrobials that have been compromised by resistance. Together, each of these antimicrobial resistance mechanisms is important for fundamental physiological studies in bacteria, for reversing the prevalence and incidence of drug-resistant pathogens in food production environments in agriculture and for the restoration of clinical efficacy in human healthcare.

## 3. Use of Antimicrobials in Food Production Environment

### 3.1. Historical Perspective

The discovery of the first antibiotic penicillin in 1929 by Alexander Fleming paved the way for the modern antibiotic era [57]. The discovery of several antimicrobials soon followed, such as sulfanilamide (1932) [58], streptomycin (1942) [59], bacitracin (an aminoglycoside) (1945), chloramphenicol (phenicols) (1947), chlortetracycline (tetracyclines) (1948), erythromycin (macrolide) (1952), vancomycin (glycopeptides) (1953), cycloserine (1955), rifampicin (rifamycins) (1957), ciprofloxacin (quinolones) (1961) and several others [60]. The primary driving force for the discovery of antimicrobial agents was the fight against the infectious disease–causing agents in humans and the application of antimicrobials for similar purposes in animals. However, an unexpected effect of antimicrobial application was the enhancement of the growth of food animals. This observation changed the perspective of antimicrobial use in food animals, leading to controversy between the essentiality of their applications in human medicine and the economic need for their use in food animals.

The positive effect of antibiotics on growth was first observed in chickens that were fed with fermentation by-products of the tetracycline-producing *Streptomyces aureofaciens* bacterium in the 1940s [61]. The growth-promoting effect of streptomycin also prompted its use in farm animal feed [62]. Subsequently, several antimicrobial agents such as chlortetracycline, folic acid, carbadox, tilmicosin, tylosin, etc., were employed as antibiotic growth promoters (AGPs) in food animals (Table 1). The growth-promoting activities of antibiotics are based on the observed effects of antibiotics on animal growth, and no scientific study has conclusively demonstrated the exact mechanism(s) by which the antibiotics lead to growth enhancement. It is hypothesized that antibiotics fed to the animals interact with the physical environment in the intestinal tract and the associated microflora, leading to positive effects on the growth of the farm animal. Some plausible mechanisms include (i) clearing of chronic bacterial infections and general improvement of animal health; (ii) improved colonization of the intestine by beneficial bacteria; (iii) increased production of vitamins and other growth factors; and (iv) thinning of the intestinal cell wall leading to better absorption of nutrients. Perhaps all of these factors may be acting in synergy, leading to growth promotion in antibiotic-fed farm animals [63].

### 3.2. Current Trends in the Use of Antimicrobials in Food Production Environments

**Figure 1:**
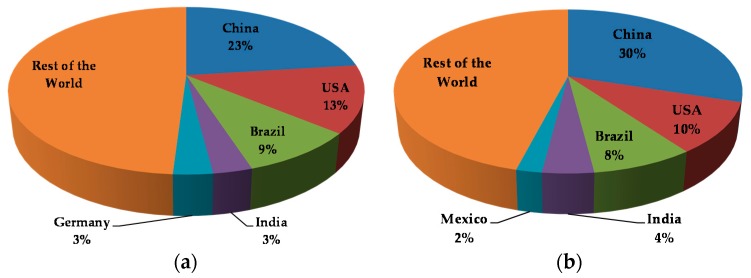
Global antimicrobial use in food animals (**a**) Major users of antimicrobials in livestock in 2010; (**b**) Major users of antimicrobials in livestock in 2030 (projected) [65].

According to one estimate, globally 63,200 tons of antibiotics were used in livestock in 2010, and by 2030, the consumption is estimated to increase by 67%, to 105,600 tons [65]. China, the USA, and Brazil are currently the leading users of antimicrobials in food animals (Figure 1a), and with the increase in the population of livestock animals, it is anticipated that the use of antimicrobials too will increase by several folds in the near future (Figure 1b). According to an estimate in 2012, about 72.5% of 12,272 tons of medically important antibiotics was used in animals, while 27.5% was used in humans in the United States [11,66]. Of the 41 antibiotics approved by the United States Food and Drug Administration (FDA) for use in animals, 31 are important for human treatment (Table 2). Over the last four decades, several antibiotics have been employed in animals for three main purposes: treatment of infections (therapeutic use), prevention of disease outbreaks in animals (prophylaxis) and growth promotion [67]. Antimicrobial treatment is advocated in animals when they are infected by a bacterial pathogen, and therapy is useful in containing the spread of disease to other members of the group and in reducing fecal shedding of the pathogen. Apart from treatment of infections, antibiotics are also used in animals as a precautionary measure to prevent the outbreak of diseases even when the animals are healthy and no infectious agent is known to exist in the rearing environment. Finally, the most controversial application of antibiotics in animal husbandry is their use as antibiotic growth promoters (AGP). Antibiotics are fed to the animals via feed in low or sub-therapeutic concentrations, and 75%–90% of the antibiotics administered this way are lost to the environment via urine and feces of the farm animals [68]. All these have direct and indirect effects on the health of humans, and as a result, the application of antimicrobials as growth promoters has been a subject of intense debate and policy-making.

Several studies have suggested that the use of antibiotic growth promoters has positive effects on the overall growth and well-being of food animals subjected to intensive farming practices [71]. Studies have reported improvements in growth rate of 4%–8% and feed conversion of 2%–8% in pigs fed with medicated feed [64,72]. In chickens, the use of antimicrobials, such as bacitracin, penicillin, chlortetracycline, oxytetracycline, erythromycin, tylosin, virginiamycin, lincomycin, bambermycin and carbadox improved weight gain and feed efficiency in broiler chicks [73]. The quantity of AGPs used varied depending on the antimicrobial, but the concentrations were generally sub-therapeutic. For example, the recommended level for the glycopeptide antibiotic avoparcin in the European Union was 20 mg/kg in pigs and 40 mg/kg in chicks [74]. However, considering the number of farm animals, the quantity of avoparcin used was several-fold higher than that used in humans. According to one estimate, 24,000 kg of active avoparcin was used in Denmark in animals compared to the 24 kg used in humans [75]. Ionophores are the second largest group of antibiotics used in farm animal production, especially in poultry for the control of coccidiosis [76]. Ionophores are considered not important from human treatment point of view, since these are never used in human medicine. Several ionophore antibiotics are used as growth promoters in animal feed such as monensin in cattle and salinomycin in swine [64].

From the animal welfare point of view, the use of antibiotics improves the general health of such animals and the hygiene of farming environments. Medicated feeds, apart from their effect on growth, have helped the animal industry to control several serious infectious diseases associated with farm animals and birds [77,78]. Sulfonamide was the first antibiotic to be used extensively in animal husbandry as a growth promoter, and this antiparasitic drug also resulted in a significant decline in the parasitic diseases of animals [71]. Although the antimicrobial use in animals resulted in a significant increase in production, a reduction in infectious diseases and in the overall well-being of farm animals, the use of antimicrobials also resulted in the development of antimicrobial resistance in bacteria associated with these animals [76].

## 4. Antimicrobial Use and the Development of Resistance in Food Production Environments

The use of antimicrobials in animal husbandry and the resultant development of antibiotic resistance in bacteria is recognized as a potential human health concern for various reasons, such as: (i) the antibiotic-resistant bacteria associated with animals may be pathogenic to humans; (ii) antibiotic-resistant bacteria can be easily transmitted to humans via food from farm animals; (iii) these antibiotic-resistant bacteria can spread to the environment through animal waste leading to wider dissemination; (iv) bacteria can transmit their resistance genotypes to human pathogens not associated with the animals via genetic element transfer mechanisms [79]. Several studies have shown that the regular use of antimicrobials in food animals and birds results in the development of antimicrobial resistance among commensal bacteria. A recent study involving seven European countries found a strong correlation between the levels of antimicrobials used and the resistances of commensal *E. coli* isolates towards those antibiotics in pigs, poultry and cattle [80]. The use of low-dose, sub-therapeutic concentrations in animal systems results in the selection of a resistant population of bacteria which colonize the animal surface, the gut and the rearing environment [81,82]. The effect of antimicrobial use is not restricted to the farm environment. Since a substantial quantity of antimicrobials is excreted by the animals via feces, environmental contamination with sub-lethal concentrations of antimicrobials occurs [83]. Environmental bacteria exposed to sub-lethal concentrations of antimicrobials can develop resistance, and through genetic exchange mechanisms, the resistance phenotype gets gradually disseminated among pathogenic and non-pathogenic bacteria. An environmental resistome builds up which can lead to wider dissemination of resistant bacteria [84]. The presence of antibiotics in foods consumed by humans has its own implications on antibiotic resistance development by the human gut microbiome [85]. The connection between antibiotic use and resistance development was first observed in *E. coli* isolated from the gut of chickens fed with streptomycin [86]. Some of the important pathogens of particular consideration with respect to their development of resistance to the above three classes of drugs include *Clostridium difficile*, *Campylobacter*, *Salmonella*, *Shigella*, and *S. aureus*. Currently, several antimicrobials of human medical use are being used in livestock, poultry, and aquaculture (Table 2). Some of these such as the fluoroquinolones are critical for the treatment of Gram-negative infections in humans, and their use in food animals may jeopardize the efficacy of these antibiotics due to the development of resistance in pathogens of zoonotic potential [87].

Scientifically based investigations suggest a strong relationship between antibiotic use in food animals and the development of antibiotic resistance by bacteria associated with farm animals [88,89,90]. There are numerous documented instances in which the introduction of an antibiotic as an AGP or for the treatment of specific infections resulted in the emergence of antibiotic-resistant bacterial variants [91]. Food animals and workers on farms using AGPs harbor more antibiotic-resistant bacteria compared to those from farms not using AGPs [92]. Antibiotic-resistant bacteria associated with food animals has resulted in their dissemination via the food chain and into the environment, and in farm workers being the carriers of such bacteria [81,93]. A study in the USA revealed that poultry workers had a 32-times-higher risk of carrying gentamicin-resistant *E. coli* compared with community referents [94].

It has been experimentally shown that the use of avoparcin in chicks resulted in the development of vancomycin resistance by *Enterococcus faecium* (VRE), and such bacteria could be transmitted to humans via their consumption of meat contaminated with VRE [74]. Prohibition of avoparcin in European Union countries in 1997 resulted in a decrease of VRE in poultry and in the community [95]. The decrease in the use of tylosin during 1998 and 1999 was followed by a decrease in erythromycin resistance among *E. faecium* and *E. faecalis* isolates from pigs [96]. An increased occurrence of virginiamycin resistance among *E. faecium* isolates in broilers correlated with the increased use of virginiamycin during 1995 to 1997 in Denmark, and the subsequent ban on the use of virginiamycin in 1998 resulted in a decrease in the resistance [96]. A strong correlation between the use of sub-therapeutic concentrations of tetracycline, alone or in combination with sulfamethazine, and the carriage of resistant strains of *Campylobacter* species in feedlot cattle has been demonstrated [97]. Quinolonesand third and fourth generation cephalosporins are important in treating salmonellosis in humans, and their widespread use in animal husbandry has selected for resistant strains that can potentially jeopardize their human clinical application [98]. A surveillance study in the USA revealed a strong association between the rise in ciprofloxacin resistance in *Campylobacter* spp. and the use of fluoroquinolones such as sarafloxacin and enrofloxacin in food-producing animals [99]. A sharp rise in ciprofloxacin resistance in *Campylobacter* as a result of the use of fluoroquinolones in food-producing animals has been reported from Denmark, Spain and the Netherlands [88,100]. The introduction of fluoroquinolones for the treatment of respiratory diseases led to the emergence of fluoroquinolone-resistant *Campylobacter* [88,101]. The use of third generation cephalosporins led to an increase in the number of cephalosporin-resistant *Salmonella* strains [102]. A study by Alexander et al. [103] has shown that sub-therapeutic administration of tetracycline in combination with sulfamethazine increased the prevalence of tetracycline- and AMP-resistant *E. coli* in cattle. The development of multidrug resistance in *E. coli* and *Salmonella* in swine fed with antimicrobial feed has been reported by several investigators [104,105,106,107]. Similarly, it has been reported that macrolides, which are widely used in food animals, result in the selection of macrolide-resistant *Campylobacter* spp. [98]. Considering the importance of macrolides in the treatment of *Campylobacter* infections in children, its use in food animals requires critical rethinking. Another serious pathogen associated with livestock and which poses the danger of transmission via food-producing animals is the methicillin-resistant *Staphylococcus aureus* (MRSA). A particular sequence type, ST398, of livestock-associated MRSA (LA-MRSA) has also been found in farm facilities and in farm workers [108]. However, there is no evidence to directly link human infections of LA-MRSA to their livestock origin.

Historically, when antimicrobial agents were first used in agriculture, such as in food production environments, bacterial multidrug-resistant variants of *S. enterica* and *E. coli* soon emerged [109,110]. Shortly after the emergence of this multidrug resistance occurred, transfer of the resistance determinants was detected in the form of plasmids, thus facilitating their movement from food animals to humans and the subsequent transmission to the gastrointestinal tract of new human hosts [91,111]. Together, mobile genetic transfer elements (e.g., bacteriophages, plasmids, gene cassettes, integrons, transposons) and various modes of infection transmission (e.g., direct food animal to human and food-borne) have facilitated the spread of newly emerged resistance determinants from food animals and their food products to humans and their communities, as a consequence of exposure to sub-lethal concentrations of antimicrobial agents [112,113]. These sub-inhibitory concentrations of antimicrobial agents used in agriculture exert selective evolutionary pressures upon the populations of bacteria that reside in food production environments, resulting in the emergence and subsequent predominance of multidrug-resistant variants [52,114]. Such multidrug-resistant variants have included Gram-positive and –negative bacteria such as *Enterococcus* spp. [115], *Lactococcus* spp. [116], *Lactobacillus* spp. [117], *S. aureus* [118], and Gram-negative bacteria such as *E. coli* [119], *Campylobacter* spp. [120], *Listeria monocytogenes* [121] and *S. enterica* [54,122]. Consequently, it has since been widely documented that antimicrobial use in food animals correlates not only with the emergence of bacterial multidrug resistance but also with the spread of infectious diseases that are recalcitrant to efficacious chemotherapy in humans [92,114,123,124].

Policy practices implemented in Europe in which antimicrobial non-therapeutic uses are completely banned (e.g., avoparcin and virginiamycin) in agriculture settings have resulted in reductions in both the prevalence of multidrug-resistant pathogens and in the minimal inhibitory concentrations (MICs) for clinically relevant antimicrobials, such as vancomycin [92,124]. Despite the extensive amount of work over a span of 50 years definitively demonstrating an association between antimicrobial agents and the incidence and prevalence of multiple drug-resistant microorganisms in food production circles [125], the issue remains surprisingly controversial in agriculture, but apparently not in human healthcare settings. Therefore, with respect to antimicrobial usage in agriculture, more work will be necessary to gain a fuller understanding of the nature and extent of the resistance prevalence problem, the overall efficacy of the antimicrobial bans, and the resulting improvement in human health [92]. A review study by the European Food Safety Authority (EFSA) and the European Medicines Agency (EMA) on the impact of measures taken to reduce the use of antimicrobials in food-producing animals on the occurrence of antimicrobial resistance (AMR) in bacteria from food-producing animals suggested an association between the reduction in antimicrobial use and reduced AMR [126], although a multiplicity of factors contribute to AMR development. An integrated approach involving different stakeholders to reduce the use of antimicrobials in animals has been recommended, which emphasizes better management practices to reduce the risks of disease, searching for alternative strategies such as competitive exclusion of pathogens, immunostimulants, probiotics, and bacteriophages, and avoiding the preventive use of antimicrobials, etc. [126].

## 5. Conclusions

Although the actual impact of the effect of antimicrobial use and the resultant resistance development by human pathogenic bacteria is a topic of intense debate [127,128], the worldwide concern of the rapid emergence of antibiotic resistance in human pathogens has led to the banning of antimicrobial use for growth promotion in animals by many countries. Considering the potential link between the use of antibiotics and their effect on human health, the World Health Organization (WHO) recommended the termination of AGPs belonging to classes of antibiotics used in human treatment and cautious application of other AGPs with routine monitoring of resistance development [129], emphasizing the need for better health management of animals and avoidance in the use of antimicrobials as growth promoters and prophylactics. The WHO report on the surveillance of antimicrobial resistance in bacteria associated with animal husbandry including livestock, poultry and aquaculture has opined that the antibiotic-resistant bacteria emerging in animal husbandry can potentially spread to human populations [130]. A joint expert meeting by Food and Agricultural Organization (FAO)/World Health Organization (WHO)/World Organization for Animal Health (OIE) held in Rome, Italy, called for the prudent use of antibiotics and a holistic approach to the control of the spread of antimicrobial resistance by minimizing the use of antimicrobials in animal husbandry [131]. Vaccination of animals, prevention of pathogen entry, and maintenance of general health and hygiene can help in reducing the use of antimicrobials in animals. Quinolones, third and fourth generation cephalosporins, and macrolides are considered the top three critically important antimicrobials from the risk assessment point of view owing to their importance in human medicine [131]. The use of antimicrobials in farm animals should be restricted to the prevention and treatment of infections under the supervision of veterinary experts. Several countries are taking proactive measures to curtail the use of antibiotics in animal husbandry and encourage their prudent use whenever they are required and under strict monitoring. Denmark and Sweden were the first to implement regulations on antibiotic use in animals and their experiences have shown positive impacts on the reduction of antimicrobial resistance in bacteria with minimum economic implications to the industry [132]. In the absence of antibiotic applications, animal health was maintained with good farming practices with no decreases in farm outputs or overall performances. Some countries have banned or levied stringent restrictions on the importation of meat subjected to antibiotic treatment on farms or during their processing such as in the antibiotic rinsing of processed poultry [133,134]. In European Union countries, antibiotic use in farm animals requires prescriptions [135], and along similar lines, the FDA has made a prescription-based administration of its new class of veterinary feed directive (VFD) antibiotics [136].

The lag in the discovery or the development of new antimicrobial drugs is worrisome in the face of failing current antimicrobial therapy regimens. The therapeutic efficacy of old and forgotten drugs is being retested, and some of them are being modified to restore their therapeutic efficacy. In this context, the use of antimicrobials in animal systems should carefully be judged from the perspective of its direct or indirect impact on human health and the environment. Considering the global nature of the antibiotic resistance problem, concerted global efforts are necessary to promote the prudent and responsible use of antibiotics in animals with constant monitoring of resistance development in these systems and the potential transfer of such bacteria throughout the food chain. It is critically important to follow the recommendations of the WHO [89], especially with respect to antimicrobials identified as critically important in human medicine, in order to prevent future crisis in the treatment of infectious diseases.

## Figures and Tables

**Table 1:** Antibiotics used for growth promotion and feed efficiency in food animals [64].

Antibiotic Group	Antibiotic(s) Used as Growth Promoters
Glycolipids	Bambermycin, avoparcin, ardacin
Streptogramins	Virginiamycin
Oligosaccharide	Avilamycin
Polypeptide	Bacitracin
Ionophore	Monensin, salinomycin
Macrolide	Tylosin, spiramycin, erythromycin
Tetracycline	Chlortetracycline, oxytetracycline
Quinoxalines	Carbadox, olaquidox
Elfamycin	Efrotomycin
Pleuromutilins	Tiamulin
β-Lactam	Penicillin

**Table 2:** Antimicrobial classes important in human medicine used in animals [69].

Antibiotic Class	Examples of Antibiotics Used in Animals	Importance in Human Medicine *
β-lactams	Penicillin, amoxicillin, ceftiofur	Critically important
Macrolides and lincosamides	Erythromycin, tylosin ^$^, tilmicosin ^$^, tulathromycin ^$^, lincomycin ^$^	Critically important
Aminoglycosides	Gentamicin, neomycin	Critically important
Fluroquinolones	Ciprofloxacin, enrofloxacin, danofloxacin ^$^	Critically important
Tetracyclines	Tetracycline, oxytetracycline, chlortetracycline	Highly important
Sulfonamides	Several sulfonamides and sulfonamide derivatives	Important
Streptogramins	Virginiamycin ^$^	Highly important
Polypeptides	Bacitracin	Important
Phenicols	Florfenicol ^$^	Highly important
Pleuromultilin	Tiamulin ^$^	-

* Classification of antibiotics based on World Health Organization expert consultation on critically important antibiotics [70]; ^$^ Veterinary use only.
